# An equal start: absence of group differences in cognitive, social, and neural measures prior to music or sports training in children

**DOI:** 10.3389/fnhum.2014.00690

**Published:** 2014-09-09

**Authors:** Assal Habibi, Beatriz Ilari, Kevin Crimi, Michael Metke, Jonas T. Kaplan, Anand A. Joshi, Richard M. Leahy, David W. Shattuck, So Y. Choi, Justin P. Haldar, Bronte Ficek, Antonio Damasio, Hanna Damasio

**Affiliations:** ^1^Brain and Creativity Institute, Dornsife College of Letters, Arts and Sciences, University of Southern CaliforniaLos Angeles, CA, USA; ^2^Thornton School of Music, University of Southern CaliforniaLos Angeles, CA, USA; ^3^Signal and Image Processing Institute, Ming Hsieh Department of Electrical Engineering, University of Southern CaliforniaLos Angeles, CA, USA; ^4^Ahmanson-Lovelace Brain Mapping Center, Department of Neurology, University of California Los AngelesLos Angeles, CA, USA

**Keywords:** music training, social and emotional skills, brain development, empathy, neuroimaging (anatomic)

## Abstract

Several studies comparing adult musicians and non-musicians have provided compelling evidence for functional and anatomical differences in the brain systems engaged by musical training. It is not known, however, whether those differences result from long-term musical training or from pre-existing traits favoring musicality. In an attempt to begin addressing this question, we have launched a longitudinal investigation of the effects of childhood music training on cognitive, social and neural development. We compared a group of 6- to 7-year old children at the start of intense after-school musical training, with two groups of children: one involved in high intensity sports training but not musical training, another not involved in any systematic training. All children were tested with a comprehensive battery of cognitive, motor, musical, emotional, and social assessments and underwent magnetic resonance imaging and electroencephalography. Our first objective was to determine whether children who participate in musical training were different, prior to training, from children in the control groups in terms of cognitive, motor, musical, emotional, and social behavior measures as well as in structural and functional brain measures. Our second objective was to determine whether musical skills, as measured by a music perception assessment prior to training, correlates with emotional and social outcome measures that have been shown to be associated with musical training. We found no neural, cognitive, motor, emotional, or social differences among the three groups. In addition, there was no correlation between music perception skills and any of the social or emotional measures. These results provide a baseline for an ongoing longitudinal investigation of the effects of music training.

## INTRODUCTION

Observing a young child in a choir, singing a melody with perfect pitch, or another at a recital translating musical notations into precisely timed finger movements on a small violin, it is natural to wonder whether traits that might precede training favor musical abilities and even motivate children to pursue music training instead of other activities. For example, are children with pre-existing superior listening skills more likely than others less gifted to seek participation in music training programs? Or is it the case that the better listening skills found in adult musicians can be fully explained by their long-term regular and intensive training in music? In either case, the difference between musically gifted or merely competent playing can be related, at least in part, to differences in measureable aspects of cognitive, motor, and emotional performance, as well as neural structure and function.

Playing music is a complex task that of necessity engages many different brain regions because it requires the concurrent recruitment of distinct sensory systems, including the auditory, somatosensory, and visual, as well as the interplay of these sensory systems with the motor, executive and affective systems. The mastering of this rich and demanding process requires regular and intense practice, and it involves coordinating both hands, and communicating emotionally with other players and listeners. The combination of such demands is likely to influence the development and maintenance of brain structures and their function.

Accordingly, over the past two decades, several investigators have reported differences in the brain and behavior of musicians compared to non-musicians (For comprehensive reviews please see Gaser and Schlaug, 2003; Herholz and Zatorre, 2012; Levitin, 2012). Music training, as one might expect, has been found to be positively associated with superior performance on a variety of auditory tasks including frequency discrimination (Schellenberg and Moreno, 2010), perception of pitch in speech (Schön et al., 2004; Wong et al., 2007), detection of minor changes of pitch in familiar (Schellenberg and Moreno, 2010) and unfamiliar melodies (Habibi et al., 2013), identification of a familiar melody when it is played at a fast or slow tempo (Andrews et al., 1998; Dowling et al., 2008), and recognition of whether a sequence of chords ends correctly based on Western classical music rules (Koelsch et al., 2007).

Music training has also been reported to have positive associations with cognitive domains in both adults and children that are only indirectly related to music, namely language skills including phonological awareness (Moreno et al., 2009, 2011; Degé and Schwarzer, 2011), vocabulary (Piro and Ortiz, 2009; Forgeard et al., 2014), verbal memory (Franklin et al., 2008; Jakobson et al., 2008), visuospatial abilities (Stoesz et al., 2007; Patston and Tippett, 2011), mathematical skills (Bahr and Christensen, 2000; Vaughn, 2000; Haimson et al., 2011), IQ (Schellenberg, 2004, 2006, 2011a), and overall academic achievement in children (Fitzpatrick, 2006; Schellenberg, 2006). On the other hand, despite strong evidence supporting cognitive benefits from music training as discussed above, a number of studies have failed to find such associations (Bilhartz et al., 1999; Costa-Giomi, 1999; Mehr et al., 2013). It is possible that differences in length or method of music training may produce different effects and explain some of these inconsistencies.

Turning to the brain itself, differences between musicians and non-musicians have been found, predictably, in auditory (Schneider et al., 2002; Gaser and Schlaug, 2003; Tillmann et al., 2003; Zatorre, 2005; Bangert and Schlaug, 2006; Jäncke, 2009; Herholz and Zatorre, 2012), and sensorimotor areas (Schlaug, 2001; Gaser and Schlaug, 2003; Jäncke, 2009). Of note, structural differences have also been reported outside of auditory and sensorimotor-related regions such as in the inferior frontal regions (Sluming et al., 2002) and multimodal integration regions (Münte et al., 2001; Bangert and Schlaug, 2006; Zatorre et al., 2007), suggesting that music-related neuroplasticity extends to brain regions that are not primarily engaged in processing music by immediate sensorimotor demands.

Despite the increasing interest in the benefits of music training and in the differences found in the brain and behavior of musicians and non-musicians, the interpretation of the findings remains unclear. For example, the differences reported in cross-sectional studies, which mostly employ quasi-experimental designs, might be due to long-term regular and intensive training or might result primarily from pre-existing biological factors that would predispose an individual to develop musical aptitude if exposed to music during a sensitive period of development. The differences also may result from contributions of both training and pre-existing factors, with certain differences possibly relating more primarily to training and others more primarily to pre-existing factors.

Learning and practicing new skills other than music have been previously shown to correlate with changes in the structure and function of the brain (Maguire et al., 2000; Golestani et al., 2002; Draganski et al., 2004; Boyke et al., 2008). Instrument-related anatomical changes in musicians provide specific indication for the role of training – for example Bangert and Schlaug (2006) reported that the amount of motor cortex devoted to the hand is larger in pianists than for non-musicians in both hemispheres, whereas in violinists the enlargement of the same region applies only to the right hemisphere which controls the left hand. This fine anatomical difference is possibly related to the fact that pianists use both hands while playing (intense bimanual finger movements), whereas violinists primarily use their left hand to manipulate the strings and the right to control the bow (unilateral intense finger movement). Likewise, enhanced auditory evoked potential responses (P200) to the instrument of practice have been shown in children after only 1 year of music training (Shahin et al., 2004). These findings lend support to the idea that the differences between musicians and non-musicians could be parsimoniously explained by long-term training in a specialized task, musical or not. Nonetheless, the experimental design in the studies reviewed above does not allow us to exclude the possibility that selection differences between the groups may be responsible for the findings. Certainly the possibility that pre-existing biological differences that precede training may contribute significantly to musical expertise has not been excluded.

Another much-debated question in relation to music training is the extent to which benefits for non-musical abilities are strictly cognitive or extend to emotional and social skills. Music is intimately connected with emotion and feelings (Juslin et al., 2008; Hunter and Schellenberg, 2010; Habibi and Damasio, 2014), and can evoke a broad range of emotions from joy and peacefulness to sadness and fear. Music also has an enormous ability to connect people with each other but the neural correlates of this connection are not completely understood (DeNora, 2010; Juslin and Timmers, 2010). Therefore, it is reasonable to expect that involvement with music can improve emotional and social abilities, although to date, the available evidence is negative, or at least inconclusive. In a recent study Schellenberg (2011b) showed that adults with at least 8 years of private music lessons, despite having a higher IQ compared to their untrained counterparts, did not show an advantage in overall emotional intelligence. Similarly, in a study with a large sample of elementary school children, music training was shown to be positively associated with IQ and academic achievement but not with social skills (Schellenberg, 2006). Finally, in a group of 6-year old children who were assigned to 1 year of drama, keyboard, or voice lessons, only the drama group showed significant improvements in social skills (Schellenberg, 2004; Goldstein et al., 2009). These findings seem to suggest that non-musical associations with music training may be limited to measures of cognitive abilities. However, a reasonable alternative explanation for the negative outcome between music training and social/emotional skills reported in these studies may be related to the solitary nature of private music lessons when compared to the interactive nature of the comparing activity (i.e., drama; Schellenberg, 2004; Schellenberg and Mankarious, 2012), rather than to music itself.

Performing in an ensemble, as opposed to private music tutoring, requires each musician to attend carefully to different aspects of the sound produced by the other musicians, including intonation, timing, and dynamics. It also requires each player to connect emotionally with the other musicians and to integrate the intended emotional impact with their own playing. Furthermore, it has been suggested that collective music making strengthens group cohesion by forcing participants to orient their attention to a shared temporal framework (Cross, 2008). Indeed, when empathy was measured in children after 1 year of music group interaction, the music group showed higher empathy scores than a control group (Rabinowitch et al., 2012). Similarly, Kirschner and Tomasello (2009) showed that cooperative and helpful behavior was enhanced in 4-year-olds following joint music-making. Of note, school children who participated in a musical program with a specific focus on empathy – through singing or composing songs – showed high empathy levels when compared to same-age children who did not participate in the program (Laurence, 2008).

One way of addressing the uncertainties related to the effects of music training would consist of examining children prior to the onset of their group music training, and, once training begins, compare them to children involved in equally socially interactive but non-musical training, such as a group athletics program, and follow both groups longitudinally. This article describes reports on the first year results of such a study.

### AIMS

The first aim for the initial phase of the study was to investigate whether children who participate in intense after-school musical training, compared to a group of children participating in after-school athletic training, show differences in evaluations testing cognitive, motor, and musical behavior or in structural and functional brain measures prior to their training. The athletic training group – after-school soccer playing – was selected as to control for those aspects of musical training that would be shared with such sport activity – a motivating, sustained, and engaging sensorimotor group learning activity. Both music and sports programs were community-based programs offered free of charge in neighborhoods of downtown Los Angeles and Rampart district. Participants, in both programs, enrolled voluntarily in their respective programs. All the students who signed up for the sports program were admitted, while the final selection for the music program was by a lottery admission from the list of students who had signed up. The reason for the lottery enrolment is due to the limited number of available slots per year. A second comparison group of children from the same area was recruited. In this case the intention was to recruit children not involved in any particular systematic organized after-school activity.

The second aim was to determine whether the children participating in music training, compared to the control groups, showed different emotional and socials skills at the start of the study. Furthermore, we aimed to examine whether musical abilities as measured by a music perception assessment correlate (prior to training) with emotional and social benefits (including empathy and prosocial behavior) that have been previously associated with music training in adulthood.

The third aim was to establish the base level for a 5 year longitudinal study (currently in its second year) designed to investigate the effects of early music training on cognitive, social, emotional, and neural development.

## MATERIALS AND METHODS

### PARTICIPANTS

Fifty 6- to 7-year-olds were recruited from public elementary schools and community music and sports programs in the greater Los Angeles area. Seventeen children (7 girls and 10 boys, mean age 79.9 months, SD = 6.8) were about to begin training with the Youth Orchestra of Los Angeles at Heart of Los Angeles, YOLA at HOLA, for short. The program is based on the Venezuelan approach known as “El Sistema” and offers free instruction 5 days a week to children from underserved areas of Los Angeles. The children are involved in a systematic and high intensity musical training that focuses on work on rhythm, melody, harmony, timbre, and emphasizes ensemble practice and group performances. Children enrolled in this program are selected, by lottery, up to a maximum of 20 per year, from a list of interested families. Seventeen children (5 girls and 12 boys, mean age 79.06 months, SD = 9.2) formed the first control group (control sports) who were about to begin training with a community-based soccer program and were not engaged in any musical training. This program allows all children whose parents enroll them in the program. The 17 enrolled in the study (to match the music target group) were those first 17 that showed interest in participating. The soccer program offers free and high intensity training (three times a week for 2 h each, with an addition of 1 h game each weekend) to children ages 6 and older. In addition, sixteen children (4 girls and 12 boys, mean age 81.5 months, SD = 6.47) formed the second control group (control non-sports). Children in this second control group were recruited from public schools in the same area of Los Angeles and were not involved in any systematic and intense after-school program of any kind. All three cohorts are from equally under-served minority communities including primarily Latino and Korean families, of downtown Los Angeles. All children were raised in bilingual households, but all attended English speaking schools and spoke English fluently.

One child from the music group and two children from the control sports group discontinued their participation in the program after the initial assessment and were not included in the final analysis. Additionally one child from the control non-sports group and one child from the music group relocated after completing the first part of the testing session, and could not continue to participate in the study; therefore they were not included in the final analysis. In total 45 children, 15 in each group, participated in the study (after attrition: music group *n* = 15, 7 girls, sports control, *n* = 15, 5 girls and non-sports control, *n* = 15, 4 girls).

### MATERIALS AND PROCEDURES

Study protocols were approved by the University of Southern California Institutional Review Board. Informed consent was obtained in writing, in preferred language, from the parents/guardians on behalf of the child participants and verbal assent was obtained from all individual children. Either the guardians or children could end their participation at any time. Participants (parents/guardians) received monetary compensation for their child’s participation and children were awarded small prizes (e.g., toys or stickers).

Children were tested individually, and in private, in two to three sessions, for a total of 5 h, completed over the course of 2–3 weeks. Testing sessions took place at the children’s music school or at our laboratory in the Brain and Creativity Institute at the University of Southern California. Magnetic resonance (MR) imaging and electroencephalography (EEG) sessions took place in the Dornsife Cognitive Neuroscience Imaging Center also at the University of Southern California. All structural MR images were handled according to the established policy of Dornsife Cognitive Neuroscience Imaging Center. They were sent to a neuroradiologist for review. If an incidental finding would be detected, the neuroradiologist would have contacted the physician designated specifically for that purpose by the family at the time they signed the informed consent and suggest further evaluation if needed.

The order of the children’s first behavioral test was counterbalanced across participants and subsequent tests were administered in a pseudorandom order as testing rooms and experimenters were available. Privacy was ensured during all the steps of the research project including recruitment, data collection, and analysis. All research procedures were conducted in person and in a private setting and data were labeled with a code that only the research team could link to personal identifying information.

Parents also answered an extensive structured interview on family income, education and ethnicity, perceptions of child’s academic achievement and school participation, the child’s current and previous participation in extracurricular activities including involvement in sports or music programs, and the presence of any professional artists currently living in the child’s home. The structured interview was conducted in the parents’ preferred language, English, Spanish, or Korean, by a research assistant who was a native speaker of that language.

#### Socio-economic status

Parents indicated their highest level of education and annual household income on a questionnaire. Responses to education level were scored on a 5-point scale: (1) elementary/middle school; (2) high school; (3) college education; (4) master’s degree (MA, MS, MBA); (5) professional degree (PhD, MD, JD). Responses to annual household income were scored on a five-point scale: (0) <$ 10,000 (1) $10,000–$19,999 (2) $20,000–29,999 (3) $30,000–39,999 (4) $40,000–49,999 and (5) >$50,000. A final socio-economic status (SES) score was calculated as the mean of each parent’s education score and annual income. Combination of education and average household income is considered to be a robust measure of SES.

#### Handedness

Handedness was assessed as part of the Bruininks–Oseretsky Test of Motor Proficiency (BOT 2-brief). Children were asked to write their name, throw a ball to the experimenter and kick a ball to the experimenter. Children were classified as right or left-handers if they used either left or right hand/foot for all three tasks. They were classified as mixed-handers if they used either left or right hand for only one of the tasks. In the music group, there were one left-handed boy and one left-handed girl; in the sports control group, one left-handed girl; and in the non-sports control group, three left-handed boys (see **Table 1**).

**Table 1:** Summary of participants.

	Music		Sports control		Non-sports control	
	Male	Female	Male	Female	Male	Female
Right-handed	7	6	10	4	8	4
Left-handed	1	1	0	1	3	0

#### Assessment of cognitive development - Wechsler abbreviated scale of intelligence

The Block Design, Vocabulary, Matrix Reasoning, and Similarities subtests from the Wechsler Abbreviated Scale of Intelligence (WASI-II) for children 6 years and older were administrated (Wechsler, 1999). In the block design test, the child sees a picture of a design and is asked to recreate the design using a set of blocks, while the picture remains present, using a set of blocks. In the vocabulary subtest, the child is presented with words orally and is asked to define each one. In the matrix reasoning subtest, which is considered to be a non-verbal test of reasoning, children are required to select from a group of four or six images to complete a pattern with a missing image. In the similarities subtest, children are presented with two words that represent common objects or concepts and they are required to describe how they are similar. In addition to these subtests, children were assessed with Memory for Digit Spans (forward and backward) from Wechsler Intelligence Scale for Children. In the digit span subtest, the child first listens to and repeats a sequence of numbers said by the experimenter. In the subsequent part, the child listens to a sequence of numbers and repeats them in reverse order. In both parts, the length of each sequence of numbers increases as the child responds correctly until two trials within a same level are responded incorrectly.

#### Assessment of motor development – Bruininks–Oseretsky test of motor proficiency

The brief form of the Bruininks–Oseretsky Test of Motor Proficiency, Second Edition (BOT-2-brief form) was used. The test uses 12 engaging, goal-directed subtests to measure a wide array of gross and fine motor skills for subjects 4–21 years.

#### Assessment of music and language development

***Auditory analysis test***. The auditory analysis test (Rosner and Simon, 1971) is a measure of phonemic perception. Children are orally presented with 15 words and after each word are asked to repeat the word and then to say the word again while omitting a particular part such as the beginning sound (e.g., say “steamboat”; now say it again but do not say “steam”).

***Gordon’s primary measures of music audiation***. The Gordon’s primary measures of music audiation (PMMA) requires children to listen to a recording of 40 pairs of simple rhythms and 40 pairs of tone sequences and make a same/different judgment for each pair by circling a pair of same or different faces (Gordon, 1986).

#### Assessment of emotional and social development

***Reading the mind in the eyes***. The Reading the Mind in the Eyes test for children (Baron-Cohen et al., 2001) consists of the presentation of photographs of faces with only the eyes showing. Children are required to choose the emotional and mental state term that best describes the eyes from four options. Children were encouraged to ask for the definition of any word they did not know. There are 28 items in this measure.

***Empathy: index of empathy for children***. The Index of Empathy for Children (Bryant, 1982) assesses children’s judgments of whether they have an emotional response to other’s emotional situations. Sample statements include: “It makes me sad to see a girl who can’t find anyone to play with.” “I really like to watch people open presents, even when I don’t get a present myself.” “I get upset when I see a boy being hurt.” There are 22 items in this measure and the answers are classified to yes/no option.

***Empathy: video emotion match test***. The video emotion test (Goldstein and Winner, 2012) investigates children’s emotional responses to fictional characters and situations in a movie. Children watched four short clips from movies in which the main character was either feeling sad or scared. They were then asked how they thought the main character felt (measure of theory of mind), how they themselves felt (measure of empathy), and how sorry they felt for the character (measure of sympathy/compassion).

***Pro-social behavior: helping and sharing***. The helping and sharing test was developed for this study to investigate children’s pro-social behavior. During a building game, using wooden blocks, two unexpected events take place: (1) the experimenter accidentally drops one of the wooden pieces, the expectation being that the child will pick up the piece (helping task) and (2) at the end has no more pieces left while the child still has some blocks, the expectation being that the child will share some of his/her blocks with the examiner (sharing task). The activity was videotaped and subsequently coded by a research assistant who did not take part in the experiment, using five score categories (Kirschner and Ilari, 2014).

To determine whether Gordon’s PMMA correlated with any of the social or emotional assessments, two-tailed bivariate Pearson correlation were performed and corrected for multiple comparisons on the age and SES matched samples collapsed across the three groups.

### IMAGING

Children underwent structural, diffusion, and functional MR imaging of their brain. Data collection and analysis for the structural scan is described below; functional, diffusion imaging, and EEG components of this study will be reported separately.

We designed a child-friendly protocol that included a training session prior to the actual scanning session. Children learned about the scanner by watching a video and getting acquainted with a scanning session in a mock scanner while listening to the different types of sound made by the scanner. During the actual scanning session, if children wished, one of the investigators remained in the scanner room and held the child’s hand. To assist children to remain motionless during the structural scans, they watched a video of their choice. After the scanning session, children were shown an actual image of their brain on the computer. High-resolution T1-weighted structural MRI images were acquired using an MPRAGE sequence on a 3T scanner equipped with a 12-channel head coil, with the following parameters: 1 mm × 1 mm × 1 mm resolution over a 256 mm × 256 mm × 208 mm FOV; TI/TE/TR = 800/3.09/2530 ms; flip angle = 10^∘∘^; GRAPPA acceleration factor *R* = 2. Due to the difficulty for children to remain motionless for extended periods of time, we acquired two separate shorter MPRAGE sequence instead of a single longer MPRAGE acquisition we would typically use for adults. These two images were then visually assessed for quality and motion artifacts, and were then registered and averaged. Two subjects from the music group, four subjects from the control sports group and one subject from control non-sports group were excluded from final analysis due to excessive motion artifacts. In total 38 subjects were included in the final structural brain analysis.

To analyze our structural MR images, we used the BrainSuite software^1^ (Shattuck and Leahy, 2002) which incorporates a multiple step cortical surface extraction and labeling sequence for analyzing T1-weighted MR images. This entailed the following steps: (1) the brain was extracted from the surrounding skull and scalp tissues using a combination of edge detection and mathematical morphology. Parameters for edge detection were adjusted to improve each individual mask followed by manual editing on each coronal slice to ensure optimal extraction. (2) MR images were corrected for slowly varying intensity non-uniformities (i.e., “shading” or “bias field” artifacts). Additionally, a manual interfaced bias-field correction tool was used^2^ which allowed us to choose areas of gray matter, white matter, and CSF to set the appropriate parameters. The tool then estimated a bias field by finding a smooth scaling function such that the selected points are approximately at median intensity of the corresponding tissue type. (3) Subsequently, each voxel in the corrected image was classified according to tissue type using a statistical classifier (Shattuck et al., 2001) that assigned labels representing white matter, gray matter, and CSF and pairwise combinations of these, as well as estimates of tissue fractions for each voxel. (4) A reference MRI atlas (ICBM452; Rex et al., 2003) with associated structure labels was aligned to the subject volume, providing labels for cerebrum, subcortical regions, brainstem, and cerebellum. These labels were combined with the tissue classification to automatically identify the cerebral white matter, to fill the ventricular spaces, and to remove the brainstem and cerebellum. The cerebrum mask yielded during this step was manually edited for all subjects, to ensure inclusion of all cerebral tissue, and exclusion of all non-hemispheric structures including brainstem and cerebellum. (5) A volume was produced whose boundary surface represents the surface of the gray/white boundary of the hemisphere, referred to as the inner cortical surface (or mask). Due to small segmentation errors, this surface often has unrealistic topological holes or handles; prior to tessellation, these holes and handles were identified and removed from the binary volume automatically using a graph based approach and further manually corrected when necessary (Shattuck and Leahy, 2001). (6) A tessellated isosurface of the resulting mask was extracted to produce a genus zero mesh surface based on the registered atlas labels that was subsequently split into two cortical hemispheres. The MR images were processed by a research assistant and subsequently reviewed by experienced research staff.

Using a surface to volume registration technique (SVReg, please see Joshi et al., 2007, 2012), a customized atlas was co-registered to each individual subject’s brain. For each brain, transfer of region labels from the atlas yielded labeled cortical surfaces and volumes with segmentation of individual lobes, the insula and the cingulate (separately because otherwise these structures had to be split between different lobes) in each hemisphere; in addition we obtain specific labeling for the cerebellum and the corpus callosum.

Each brain was examined after automated transfer and manual correction was applied whenever necessary due to edge mislabeling. We then determined gray matter volume, cortical thickness, and surface area, for each individual lobe, the insula and cingulate. Gray matter volume was computed using volumetric labels as well as the partial tissue fraction data generated by the BrainSuite and SVReg sequences which contains tissue fraction value corresponding to the percentages of gray matter, white matter and CSF in every voxel. Using these values, we computed the total amount of gray matter in each region of interest. Cortical thickness was defined as the linked distance between inner cortical surface (the gray/white separation line) and pial surface. This measure was computed at every vertex on the surface mesh defining the two surfaces. The vertexwise cortical thickness measure for all the vertices within a region of interest was subsequently averaged to produce the final cortical thickness value for that region of interest. Surface area was defined as the area of the pial cortical surface mesh for the particular region of interest. We also obtained the total volume (gray and white matter) for the cerebellum and total white matter volume for the corpus callosum.

## RESULTS

Children from the three groups performed within the normal range reported for all the standard behavioral measures (**Table 2**). Preliminary analysis revealed no differences in sex χ^2^(2, *N* = 45) = 1.35, *p* = 0.5, age *F*(2,42) = 1.52, *p* = 0.23 and socio-economic status *F*(2,42) = 1.36, *p* = 0.68 between the three groups, and therefore these factors were not included in any subsequent analysis. We then analyzed the age and SES matched sample data with a series of univariate ANOVAs with group as independent factor. ANOVA analysis revealed no significant (or even near significant) differences between groups on any cognitive, emotional, social, motor, or musical abilities. These findings are summarized in **Table 2**.

**Table 2:** Mean and SD for behavioral assessments by group; univariate ANOVA results for each behavioral outcome by group.

Assessment	Music, sports control, and non-sports control	ANOVA results
FSIQ	100.7 (11.5); 96.2 (8.45); 92.6 (10.5)	*F*(2,42) = 1.777, *p* = 0.18
Digit span	10.1 (2.1); 9.5 (2.8); 9.9 (2.0)	*F*(2,42) = 0.230, *p* = 0.79
Auditory analysis	10.6 (2.9); 9.5 (4); 10 (2.8)	*F*(2,42) = 0.380, *p* = 0.68
BOT motor development	57.1 (8); 57.4 (7.8); 57.5 (6.3)	*F*(2,42) = 0.008, *p* = 0.99
Gordon’s PMMA	61 (7.2); 60.9 (6.3); 60.4 (8)	*F*(2,41) = 0.097, *p* = 0.90
Pro-social behavior	2 (1.6); 2.3 (1.7); 2.53 (1.32)	*F*(2,42) = 0.522, *p* = 0.59
Mind in the eye	15.6 (4.2); 15.6 (4.7); 13.4 (4)	*F*(2,42) = 1.517, *p* = 0.23
Index of empathy	11.9 (4.1); 12.1 (2.2); 11.3 (2.7)	*F*(2,42) = 0.480, *p* = 0.62
Emotion match	0.48 (0.3); 0.49 (0.4); 0.6 (0.3)	*F*(2,42) = 0.502, *p* = 0.60

As for the correlation between Gordon’s PMMA and social tasks, including Pro-social Behavior, Emotion Match Assessment, Reading the Mind in the Eyes and Index of Empathy, the only significant correlation was between Gordon’s PMMA, and the Mind in the Eye’s test, however after Bonferroni correction for multiple comparisons, the correlation was no longer significant (**Table 3**).

**Table 3:** Intercorrelations among behavioral outcome variables for three groups (two tailed bivariate correlations).

	FSIQ	Mind in the eye	Index of empathy	Emotion match	Pro-social behavior
Gordon’s PMMA	0.2067	0.3488*	0.0535	0.1414	-0.0303
FSIQ		-0.0849	0.1939	-0.0329	-0.2271
Mind in the eye			-0.0473	-0.1763	-0.076
Index of empathy				-0.0015	-0.0154
Emotion match					0.1387

*Significant at *p* < 0.05.

**After a Bonferroni correction only correlations at *p* < 0.005 are deemed to be significant.

The values found in the three groups of children for gray matter volume, cortical thickness and surface area were within the range of previously reported measures for normal brain development in this age group (Lyall et al., 2014) and are shown in **Table 4**. We used multivariate analysis of variance (MANOVA) to test difference in gray matter volume, cortical thickness, and cortical surface area in each individual lobe, the insula and cingulate between the three groups (**Table 4D**). We used univariate analysis of variance (ANOVA) to test differences in total volume of cerebellum and corpus callosum among the three groups (**Table 5**). We did not find any differences between groups in any of the brain measures, in any of the regions assessed, as shown in **Tables 4D** and **5**.

**Table 4:** Mean and SD for brain measures by groups.

Area	Music	Sports control	Non-sports control
**(A) Measures of cortical thickness in mm (mean and SD) by group.**	
R frontal lobe	4.20 (0.16)	4.27 (0.25)	4.25 (0.16)
L frontal lobe	4.21 (0.16)	4.26 (0.22)	4.20 (0.20)
R parietal lobe	3.94 (0.21)	3.99 (0.12)	3.94 (0.17)
L parietal lobe	4.02 (0.25)	4 (0.20)	3.99 (0.21)
R temporal lobe	4.38 (0.16)	4.45 (0.13)	4.33 (0.23)
L temporal lobe	4.43 (0.18)	4.45 (0.11)	4.36 (0.17)
R occipital LOBE	3.50 (0.26)	3.51 (0.25)	3.41 (0.21)
L occipital lobe	3.52 (0.27)	3.56 (0.27)	3.43 (0.21)
R cingulate	4.10 (0.25)	4.11 (0.25)	4.07 (0.26)
L cingulate	4.12 (0.22)	4.07 (0.18)	4.10 (0.24)
R insula	5.16 (0.25)	5.18 (0.39)	5.12 (0.28)
L Insula	5.19 (0.25)	5.10 (0.28)	5.14 (0.29)
**(B) Measures of cortical surface area in cm^**2**^ (mean and SD) by group.**	
R frontal lobe	382.72 (24.19)	384.64 (48.45)	365.84 (28.90)
L frontal lobe	379.74 (24.30)	380.39 (44.22)	363.03 (26.40)
R parietal lobe	256.86 (27.84)	269.66 (32.59)	258.80 (21.27)
L parietal lobe	270.29 (20.80)	287.14 (35.48)	266.07 (21.18)
R temporal lobe	227.40 (18.54)	233.27 (31.72)	221.19 (17.70)
L temporal lobe	214.38 (15.99)	224.11 (36.01)	214.62 (18.35)
R occipital lobe	158.65 (13.73)	154.64 (14.37)	157.33 (17.74)
L occipital lobe	153.54 (11.76)	149.13 (23.93)	150.82 (20.74)
R cingulate	39.91 (6.41)	39.86 (6.43)	38.94 (4.71)
L cingulate	46.62 (3.92)	46.9 (5.44)	44.29 (5.45)
R insula	15.70 (1.38)	16.16 (2.08)	15.38 (2.36)
L insula	15.93 (1.42)	16.63 (2.25)	15.53 (2.30)
**(C) Measures of gray matter volume in cc (mean and SD) by group.**	
R frontal lobe	108.30 (8.72)	110.92 (10.56)	103.92 (9.40)
L frontal lobe	107.36 (8.35)	110.04 (10.38)	102.93 (9.43)
R parietal lobe	71.41 (8.41)	76.19 (6.25)	72.91 (6.69)
L parietal lobe	74.54 (7.12)	79.47 (8.43)	74.33 (6.23)
R temporal lobe	72.22 (5.75)	75.12 (9.33)	68.44 (7.95)
L temporal lobe	68.84 (4.84)	72.33 (10.63)	67.30 (7.08)
R occipital lobe	38.42 (3.46)	37.86 (3.99)	37.75 (4.07)
L occipital lobe	36.50 (3.77)	35.3 (4.79)	35.75 (5.64)
R cingulate	11.92 (1.93)	11.8 (1.44)	11.44 (1.19)
L cingulate	13.45 (1.43)	13.14 (1.40)	12.49 (1.51)
R insula	5.99 (0.50)	6.15 (1.02)	5.94 (0.96)
L insula	5.98 (0.64)	5.84 (1.09)	5.64 (1.20)

**Cortical thickness**	**Gray matter volume**	**Cortical surface area**

**(D) Multivariate ANOVA results for each brain outcome by group.**	
*F*(24,48) = 0.766, *p* = 0.75	*F*(28,44) = 0.928, *p* = 0.57	*F*(24, 48) = 0.963, *p* = 0.52

**Table 5:** Measures of total volume of cerebellum and corpus callosum in cc (mean and SD) by group – Univariate ANOVA results for cerebellum and corpus callosum by group.

Area	Music	Sports control	Non-sports control	ANOVA results
Corpus callosum	6.41 (0.66)	6.30 (1.03)	6.14 (0.75)	*F*(2,35) = 0.387, *p* = 0.68
Cerebellum	122.59 (12.54)	122.43 (13.77)	115.71 (12.68)	*F*(2,35) = 1.221, *p* = 0.3

A power analysis with fixed effects model and alpha level of 0.05 showed that the current sample size yields, an 80% probability to find an effect size typical of what has previously been reported for comparisons of adult musicians and non-musicians (Schneider et al., 2002; Gaser and Schlaug, 2003). We also performed multiple correlations between the cognitive assessment scores and total cortical volume but found no correlations.

## DISCUSSION

The immediate objective of the study was to determine if children beginning music training were different from children of the same age beginning sports training, or not involved in any formal training program. Students in both music and sports group had enrolled voluntarily in their respective programs; all students who sign up for the sports program are admitted, while the final group in the music program is based on a lottery admission from the list of students who have signed up. We did not find any differences in cognitive, motor, or musical ability, nor were there anatomical differences in terms of gray matter volume, cortical thickness, or surface area in any regions of interest between the three groups of children at the onset of the study. The absence of pre-existing differences between the music group and the control groups provides a foundation for the future investigation of how musical training affects the brain and cognitive/emotional development, the goal of the longitudinal study in which we are engaged. It also addresses a classic question: are the differences which have been consistently found in brain and behavior measures between adult musicians and non-musicians in the general population, present prior to training thus representing traits favorable to music education?

The likelihood that the differences between musicians and non-musicians are accounted for by experience and training is supported by evidence of brain plasticity with acquiring and practicing new skills (Maguire et al., 2000; Draganski et al., 2004; Scholz et al., 2009), and with observed brain reorganization following sensory loss in congenitally blind or deaf subjects (Roeder et al., 1999; Emmorey et al., 2003; Voss and Zatorre, 2012). Additional support for the role of training comes from the finding that the degree of structural brain differences between musicians and non-musicians correlates with intensity of musical training (Schneider et al., 2002; Gaser and Schlaug, 2003; Hutchinson et al., 2003), and that the brain areas that have been shown to be enlarged in adult musicians are largest in those musicians who began training at a young age (Pantev et al., 1998; Schlaug, 2001; Habibi et al., 2013). Unfortunately, without prior demonstration that pre-existing differences were absent, the significance of these findings remains unclear. Schellenberg (2004) randomly assigned 6-year old children to music lessons, drama lessons, or no lessons and tested them on IQ measures before and again after the training. The music group had a significantly larger increase than the drama and control groups from prior to training to post training. In a longer experiment however, in which 9-year olds were assigned randomly to piano lessons or no piano lessons, no differences in IQ were found at the end of the 3 year (Costa-Giomi, 1999), and there was no effect on the academic achievement. In our current longitudinal study we did not find any differences in cognitive abilities or neural measures between the music group and the two control groups as they began their activities. As indicated, a power analysis showed that with the current sample size we would have a 80% probability of detecting differences with the effect size typically reported in adults (Schneider et al., 2002; Gaser and Schlaug, 2003) if such differences had been present among our groups. Therefore, we can presume that differences between the three groups that may appear in the years to come are likely due to the learning, practice-related, and environmental differences in their respective activities.

The absence of pre-existing differences in our subject groups does not allow us to conclude that adult musicians and non-musicians are not different as children. We also note that the children in our study were not randomly assigned to their groups. Such a design would not have been feasible, as noted by Winner and Hetland (2000). The fact that there were no pre-existing differences between the music group and the control groups along with the fact that enrollment in the music group was determined by lottery and not by prior musical engagement provide an opportunity to lay the groundwork for a longitudinal study (now in its second year) of the effects of music training on cognitive, emotional, social, and brain development. We are aware of only one comparable longitudinal study (Schlaug et al., 2005), which reported cognitive and brain changes in auditory, and motor areas after instrumental music training in 5- to 7-year old children observed over 14 months. No follow up results have been reported for subsequent years (Hyde et al., 2009).

The second aim of this phase of the study was to determine whether children participating in music training, compared to a control group of children not engaged in music training, differ in terms of emotional and social skills at the beginning of their training. We did not find any differences in emotional or social skills in the three groups. We further aimed to determine whether musical skills, as measured by a music perception instrument, correlate with emotional and social outcomes that have been proposed to be associated with music practice, including empathy and pro-social behavior. No correlation was found between Gordon’s PMAA and the results of the social/emotional assessments performed at this time, suggesting that, at least prior to training, there are no evident relationship between musical abilities as measured by a music perception task and emotional and social skills. Our results also give some indirect support for the idea that the kinds of social and emotional skills reported in children who have studied music may be a by-product of music training (Rabinowitch et al., 2012). Alternatively, given that Gordon’s PMAA is a musical aptitude task that primarily measures perception skills and memory, using an assessment which instead focuses on interpersonal musical experience to correlate with social/emotional skills may be more suitable. Therefore, we have added a measure of entrainment to our battery for subsequent years of study.

Few studies to date have examined the association between music training and the development of emotional abilities and social skills. Given that music is often considered to be intimately linked with emotions and feelings, it is surprising that available research shows mixed findings (Schellenberg, 2004; Goldstein et al., 2009; Rabinowitch et al., 2012). Moreover, in studies that show positive associations between music training and emotional/social skills, it is unclear whether the results are related to cognitive advantages in musicians or the consequence of the participation in an engaging collective activity, or of a combination of those factors. One possibility is that the association between music training and emotional/social skills is measured with instruments that depend on cognitive capacities such as language comprehension. To address this possibility, these instruments can be replaced by measures that do not require verbal reports, such as Matched Faces (see Rabinowitch et al., 2012). An alternative explanation is that associations between music training and emotional/social skills depend on the type of music training, specifically whether the activity is individual or centered on a group collaboration (Kirschner and Tomasello, 2010). Given that playing music in a group requires musicians to play close attention to auditory and sensorimotor output as well as to the emotional state of other players, it is reasonable to expect that collective music making may improve pro-social behavior and strengthen group cohesion (Cross, 2001, 2008).

Our longitudinal study will allow us to examine the role of group training (in music and sports) on the development of emotional and social skills. The participants in the music group are training in a program inspired by the Venezuelan method known as *El Sistema*. El Sistema is believed by many to be an important mechanism of social change through music (Booth, 2011). The program offers free music training to children from underprivileged communities, through intensive and collective experiences. Students are enrolled in this program by a lottery admission process and spend 2 h per day after-school learning music, always in a group setting. Participation in ensembles, choirs, and bands is mandatory. These activities are viewed as a way for students to develop social skills (Cross, 2008; Uy, 2012). To account for the social nature of the music training program, we specifically selected a compatible motivating, intense, stimulating and collective activity as a comparison. Children participants in the control sports group are enrolled in soccer after-school program training, for up to 2 h, three times a week, with extra games on weekends. The continued study of children involved in these two activities will allow us to glean which changes in emotional and social development may be due to music training *per se* compared to collective group training in general as seen in sports.

In summary, the analysis reported here lays the groundwork for the longitudinal study of the effects of music training on childhood development as assessed by cognitive, emotional, social, and neural measures. In our groups, we found no differences in the levels of intellectual, emotional, and social performance, nor did we find differences in brain structure. The socio-economical and family environments are comparable. We hope our findings will demonstrate the value of musical education at a time when music education programs are being eliminated from standard curricula.

## Conflict of Interest Statement

The authors declare that the research was conducted in the absence of any commercial or financial relationships that could be construed as a potential conflict of interest.

## Footnotes

1 http://brainsuite.org/

2 http://neuroimage.usc.edu/neuro/Resources/bfc_correction_tool
